# Supplementary figures and images for: BNC1 inhibits the development and progression of gastric cancer by regulating the CCL20/JAK-STAT axis
Source: PeerJ. 2025 May 26;13:e19477. doi: 10.7717/peerj.19477 (PMC12121617; doi:10.7717/peerj.19477)

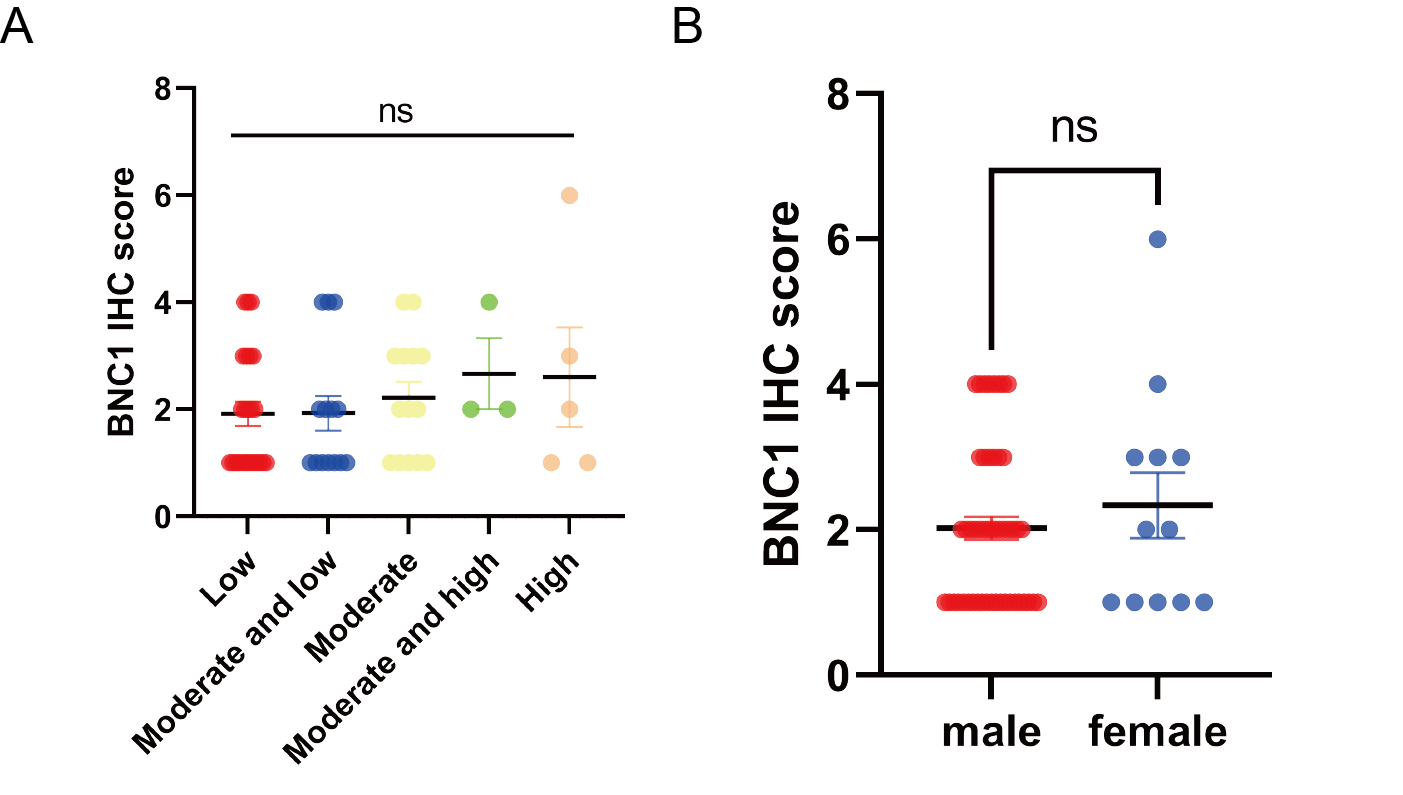

Supplement: Supplemental Information 1 — (A) Quantification of BNC1 scores in moderate and low differentiation GC tissues and high differentiation GC tissues. (n = 61). (B) Quantification of BNC1 score according to sex in GC tissues. (n = 61). [file peerj-13-19477-s001.png]

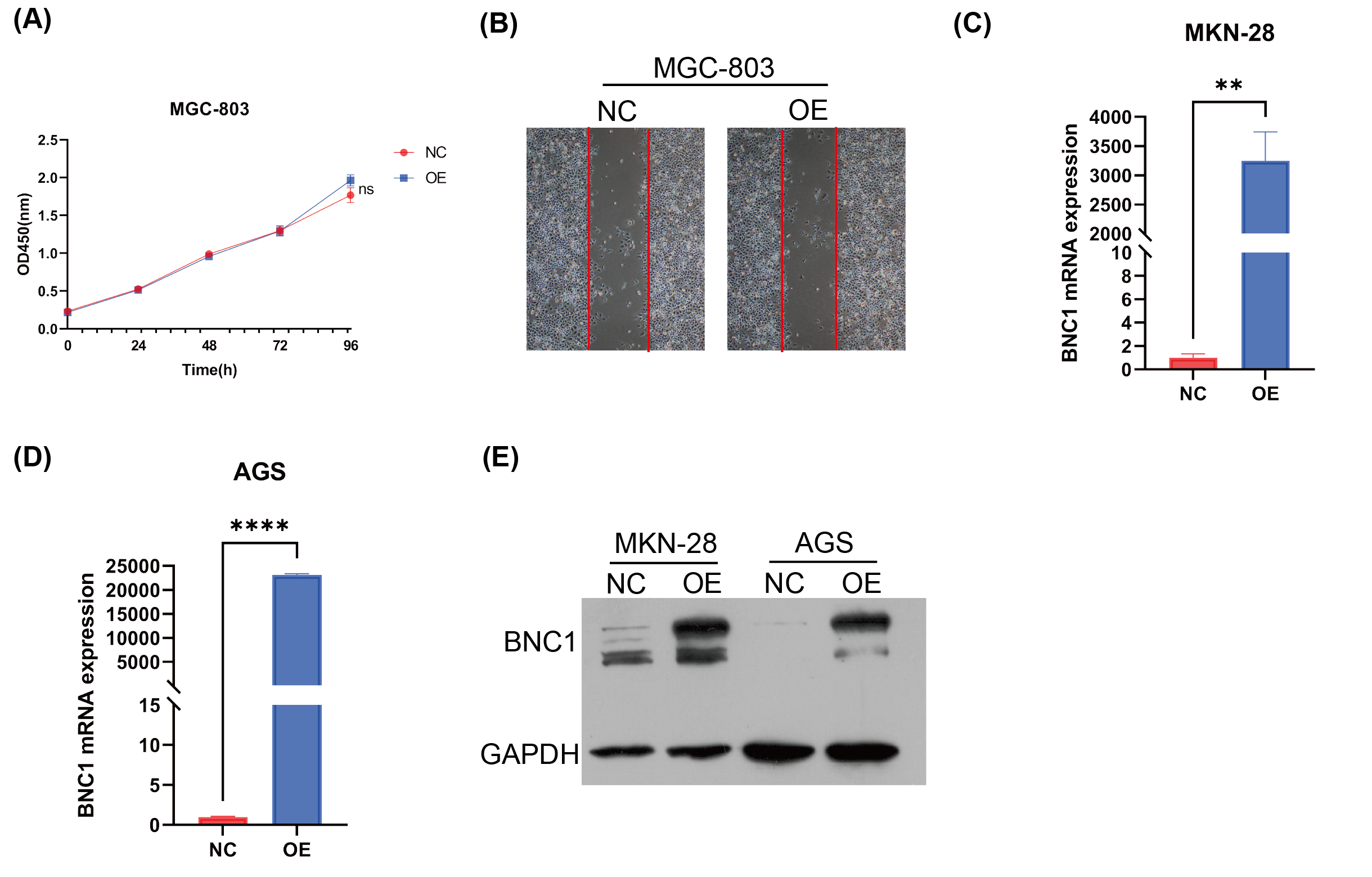

Supplement: Supplemental Information 2 — (A) CCK-8 assay of cell growth with BNC1 overexpression and control cells. (B) Wound healing assay of BNC1 overexpression and control cells. (C) RT-qPCR analysis of overexpression efficiency of BNC1 in MKN-28 cell line. (D) RT-qPCR analysis of overexpression efficiency of BNC1 in AGS cell line. (E) Western blot analysis of overexpression efficiency of BNC1 in the corresponding cell lines. [file peerj-13-19477-s002.png]

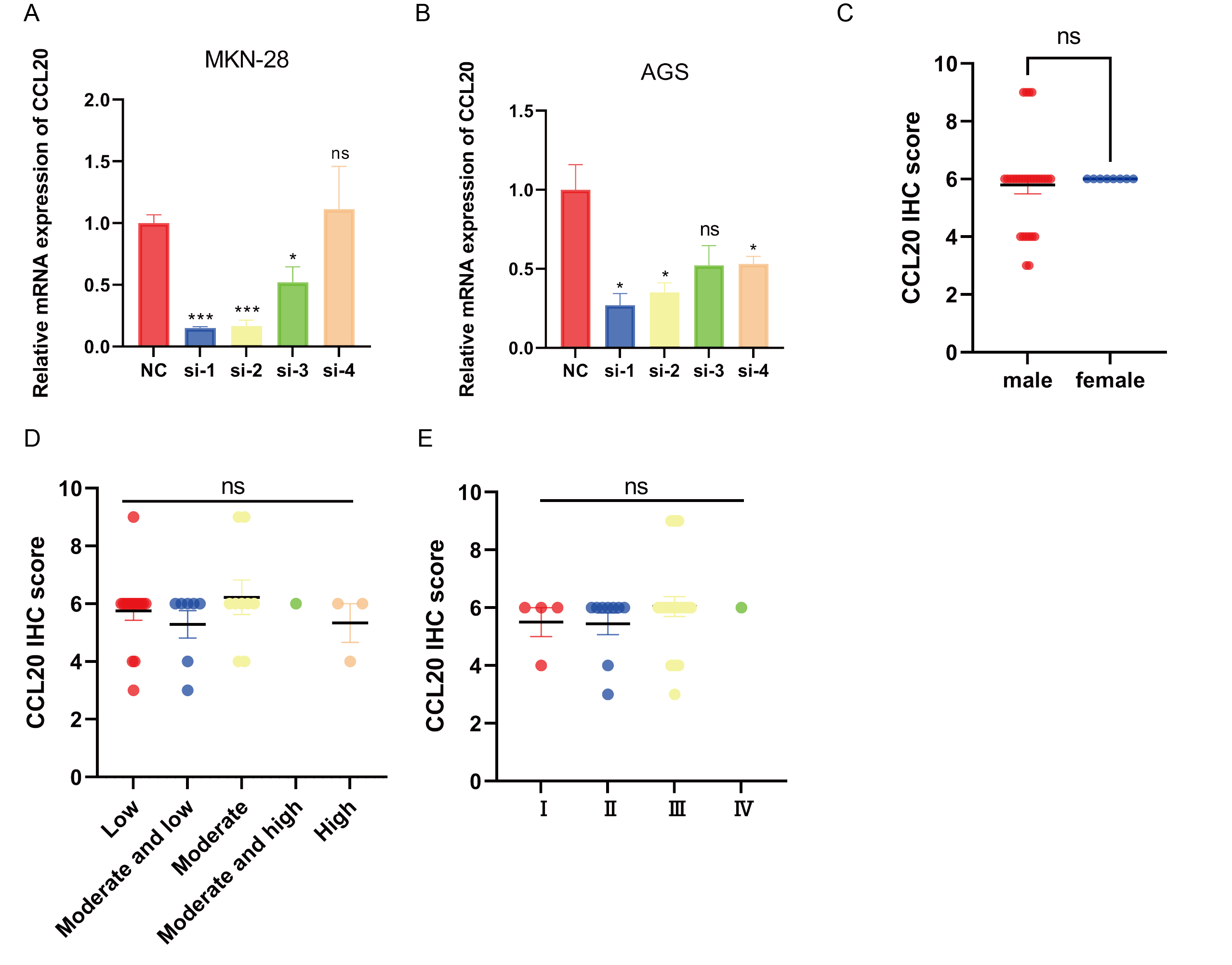

Supplement: Supplemental Information 3 — (A) qPCR validation of knockdown efficiency of CCL20 in MKN-28 cell line. (B) qPCR validation of knockdown efficiency of CCL20 in AGS cell line. (C) Quantification of CCL20 score according to sex in GC tissues. (n = 37) (D) Quantification of BNC1 scores in moderate and low differentiation GC tissues and high differentiation GC tissues. (n = 37). (E) Quantification of BNC1 scores in Stage gastric cancer tissues and - Stage gastric cancer tissues. (n = 37). [file peerj-13-19477-s003.png]

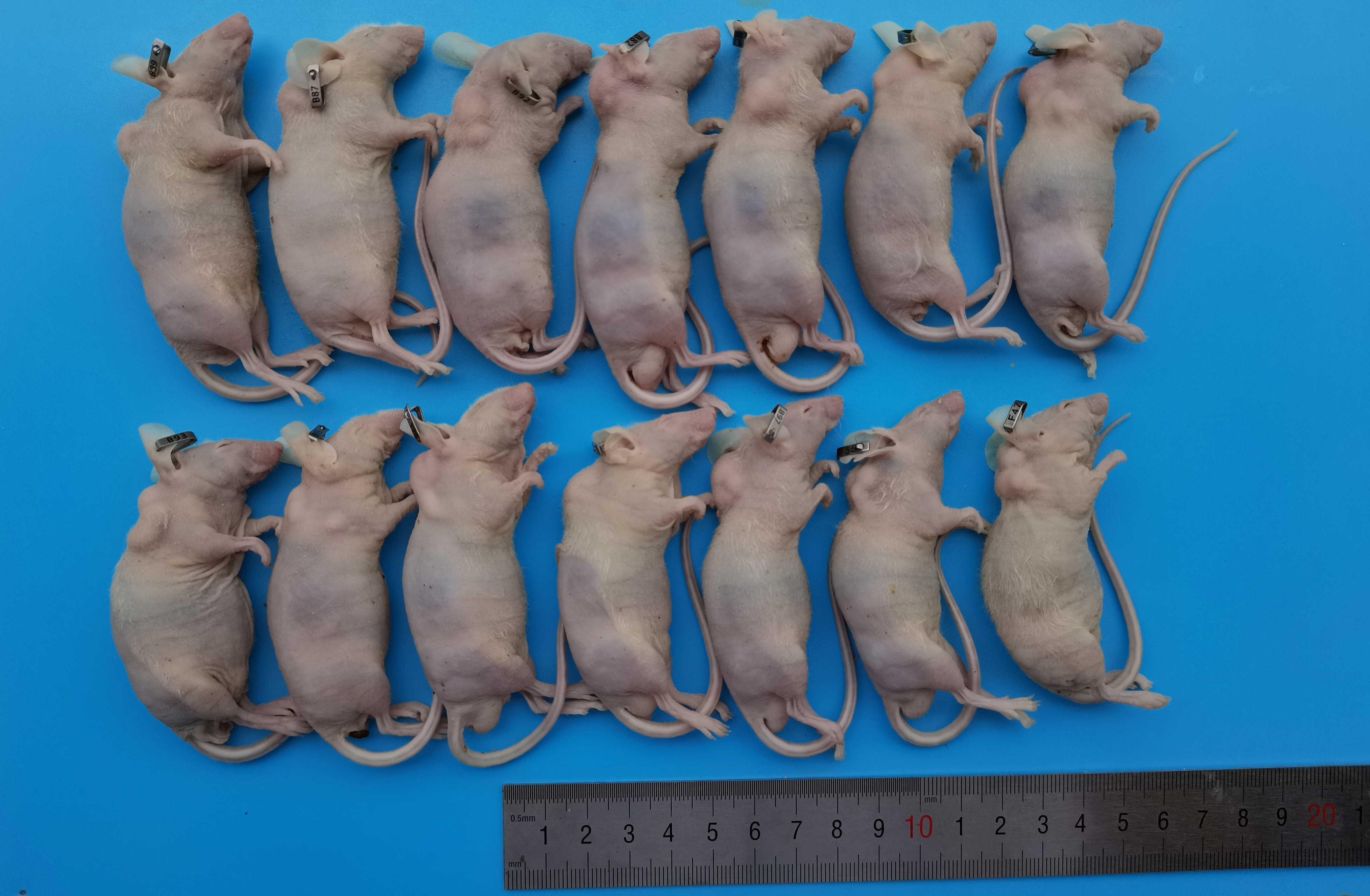

Supplement: Supplemental Information 11 [file peerj-13-19477-s011.png]

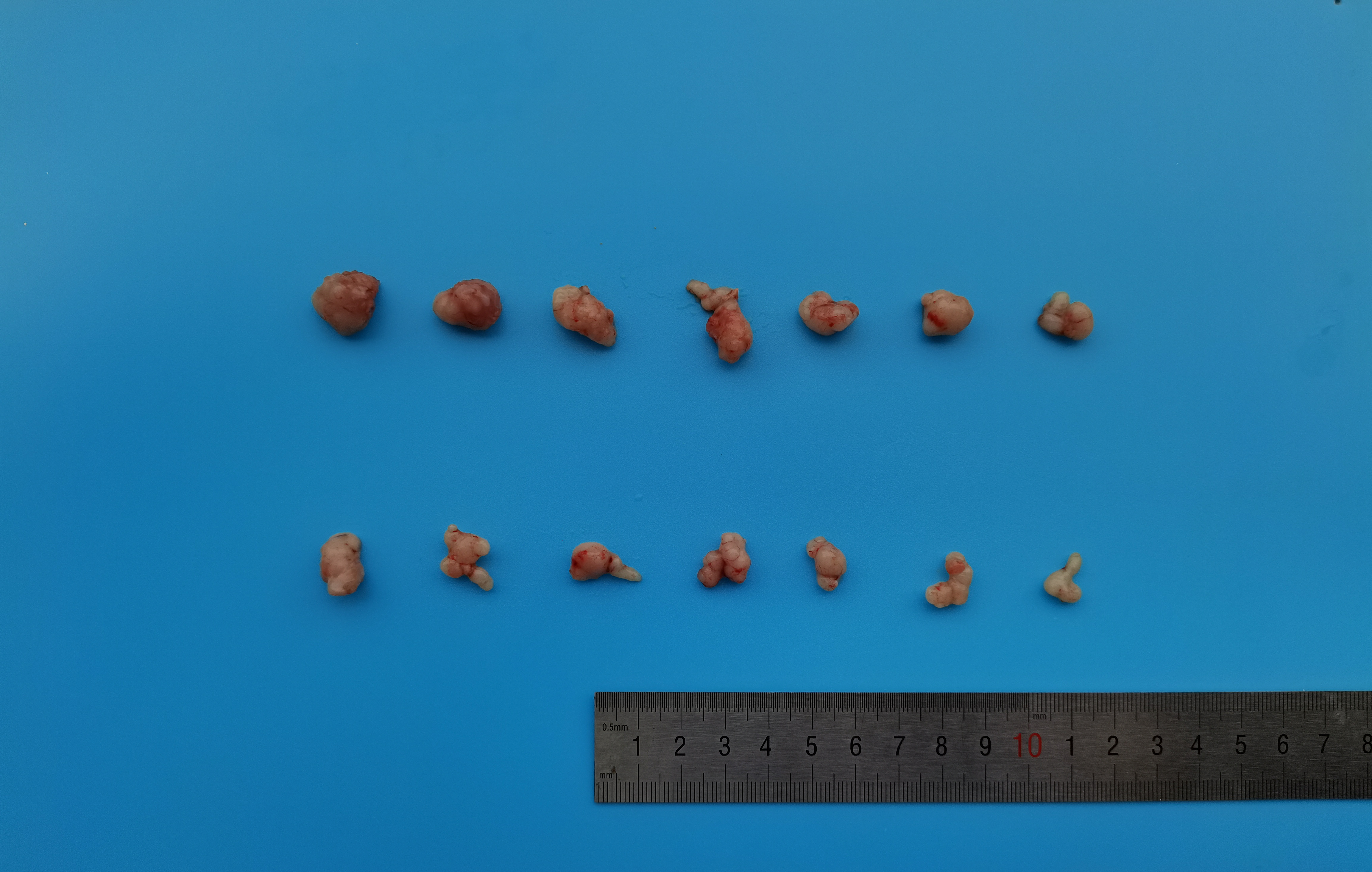

Supplement: Supplemental Information 12 [file peerj-13-19477-s012.png]
