# Supplementary material for: BNC1 inhibits the development and progression of gastric cancer by regulating the CCL20/JAK-STAT axis
Source: PeerJ. 2025 May 26;13:e19477. doi: 10.7717/peerj.19477 (PMC12121617; doi:10.7717/peerj.19477)
Supplement: Supplemental Information 5 [file peerj-13-19477-s005.docx]

**Table S2**

lentiviral and siRNA sequences

| Name | Sequence（5'-3'） |
| --- | --- |
| OE-BNC1  si-CCL20#1 | NM_001717.4  sense: CCGUAUUCUUCAUCCUAAATT |
|  | antisense: UUUAGGAUGAAGAAUACGGTT |
| si-CCL20#2 | sense: GAAGUUGAUUCAUAUUGCATT |
|  | antisense: UGCAAUAUGAAUCAACUUCTT |
| si-CCL20#3 | sense: CUUGGGUGAAAUAUAUUGUTT |
|  | antisense: ACAAUAUAUUUCACCCAAGTT |
| si-CCL20#4  si-NC and OE-NC | sense: GCUGUACCAAGAGUUUGCUTT  antisense: AGCAAACUCUUGGUACAGCTT  sense: UUCUCCGAACGUGUCACGUTT  antisense: ACGUGACACGUUCGGAGAATT |
|  |  |
