# Supplementary material for: BNC1 inhibits the development and progression of gastric cancer by regulating the CCL20/JAK-STAT axis
Source: PeerJ. 2025 May 26;13:e19477. doi: 10.7717/peerj.19477 (PMC12121617; doi:10.7717/peerj.19477)
Supplement: Supplemental Information 6 [file peerj-13-19477-s006.docx]

**Table S3**

The primer sequence of qRT-PCR

| Gene | Primer sequence (5'‑3') |
| --- | --- |
| GAPDH | F: TGCACCACCAACTGCTTAGC |
|  | R: GGCATGGACTGTGGTCATGAG |
| BNC1 | F: CAGCAGTTCCTTCGTTTTGGA |
|  | R: GGTGGTATGATGATGGATTGCTC |
| PTPRC | F: ACCACAAGTTTACTAACGCAAGT |
|  | R: TTTGAGGGGGATTCCAGGTAAT |
| SERPINB9 | F: AATGCAAGTGGTACTTTTGCCA |
|  | R: AAGCCCGATGAATGTCTTCCT |
| BANK1 | F: CCAACAGACCTACGAGCAAAA |
|  | R: ACTGCTAGTGGTATGGTGTTTCT |
| CCL20 | F: TGCTGTACCAAGAGTTTGCTC |
|  | R: CGCACACAGACAACTTTTTCTTT |
| RAC2 | F: CAACGCCTTTCCCGGAGAG |
|  | R: TCCGTCTGTGGATAGGAGAGC |
|  |  |
