# Supplementary material for: BNC1 inhibits the development and progression of gastric cancer by regulating the CCL20/JAK-STAT axis
Source: PeerJ. 2025 May 26;13:e19477. doi: 10.7717/peerj.19477 (PMC12121617; doi:10.7717/peerj.19477)
Supplement: Supplemental Information 7 [file peerj-13-19477-s007.docx]

**Table S4**

The JASPAR database was used to predict the potential binding sites of CCL20 promoter and BNC2

| Matrix ID | Name | Score | Relative score | Sequence ID | Start | End | Strand | Predicted sequence |
| --- | --- | --- | --- | --- | --- | --- | --- | --- |
| MA1928.2 | MA1928.2 BNC2 | 7.576299 | 0.8631 | 1 | 1272 | 1278 | + | tgagtta |
| MA1928.2 | MA1928.2 BNC2 | 7.5325108 | 0.8620 | 1 | 242 | 248 | + | tgagcca |
| MA1928.2 | MA1928.2 BNC2 | 7.5325108 | 0.8620 | 1 | 615 | 621 | - | tgagcca |
| MA1928.2 | MA1928.2 BNC2 | 7.2805934 | 0.8557 | 1 | 837 | 843 | - | tagaaca |
| MA1928.2 | MA1928.2 BNC2 | 7.1955614 | 0.8535 | 1 | 72 | 78 | - | tgaggca |
| MA1928.2 | MA1928.2 BNC2 | 7.1955614 | 0.8535 | 1 | 742 | 748 | + | tgaggca |
| MA1928.2 | MA1928.2 BNC2 | 6.913596 | 0.8464 | 1 | 1741 | 1747 | + | tgggtca |
| MA1928.2 | MA1928.2 BNC2 | 5.963622 | 0.8223 | 1 | 837 | 843 | + | tgtctca |
| MA1928.2 | MA1928.2 BNC2 | 5.5389504 | 0.8115 | 1 | 1272 | 1278 | - | taactca |
| MA1928.2 | MA1928.2 BNC2 | 5.3361964 | 0.8064 | 1 | 72 | 78 | + | tgcctca |
