# Supplementary material for: BNC1 inhibits the development and progression of gastric cancer by regulating the CCL20/JAK-STAT axis
Source: PeerJ. 2025 May 26;13:e19477. doi: 10.7717/peerj.19477 (PMC12121617; doi:10.7717/peerj.19477)
Supplement: Supplemental Information 8 [file peerj-13-19477-s008.docx]

**Table S3**

CCL20 immunohistochemical staining score and clinical information

| Pathology No. | Tumor differentiation | Gender | Age | T | N | M | Cell proportions score Staining intensity（Tumour） | | | Cell proportions score Staining intensity(Normal tissue) | | |
| --- | --- | --- | --- | --- | --- | --- | --- | --- | --- | --- | --- | --- |
| 2022-17196 | moderate | Female | 68 | T3 | N0 | M0 | 3 | 2 | 6 | 1 | 3 | 3 |
| 2022-24866 | low | Female | 48 | T4 | N1 | M0 | 3 | 2 | 6 | 1 | 3 | 3 |
| 2022-45755 | high | Female | 71 | T1 | N0 | M0 | 2 | 3 | 6 | 2 | 1 | 2 |
| 2022-20764 | low | Male | 70 | T4 | N0 | M0 | 3 | 2 | 6 | 1 | 3 | 3 |
| 2022-32143 | moderate | Male | 77 | T3 | N3 | M0 | 3 | 3 | 9 | 2 | 2 | 4 |
| 2022-45347 | low | Male | 59 | T3 | N2 | M0 | 3 | 2 | 6 | 2 | 2 | 4 |
| 2023-366 | low | Male | 67 | T3 | N0 | M0 | 2 | 3 | 6 | 3 | 2 | 6 |
| 2023-6411 | moderate-low | Male | 61 | T3 | N2 | M0 | 3 | 2 | 6 | 2 | 2 | 4 |
| 2023-6486 | moderate-low | Female | 81 | T4 | N3 | M0 | 3 | 2 | 6 | 2 | 3 | 6 |
| 2023-6598 | moderate | Male | 64 | T4 | N1 | M0 | 2 | 3 | 6 | 3 | 2 | 6 |
| 2023-6696 | low | Male | 65 | T4 | N0 | M0 | 1 | 3 | 3 | 1 | 1 | 1 |
| 2023-4948 | moderate-high | Male | 59 | T2 | N0 | M0 | 2 | 3 | 6 | 3 | 2 | 6 |
| 2023-5723 | moderate | Female | 65 | T2 | N2 | M0 | 2 | 3 | 6 | 2 | 2 | 4 |
| 2022-42027 | low | Female | 28 | T4 | N3 | M1 | 3 | 2 | 6 | 1 | 1 | 1 |
| 2023-3178 | low | Female | 79 | T4 | N0 | M0 | 3 | 2 | 6 | 2 | 2 | 4 |
| 2023-2894 | moderate | Male | 73 | T4 | N1 | M0 | 2 | 3 | 9 | 2 | 1 | 2 |
| 2023-12503 | moderate | Male | 74 | T3 | N2 | M0 | 2 | 3 | 6 | 2 | 1 | 2 |
| 2023-17419 | moderate | Male | 54 | T4 | N1 | M0 | 2 | 2 | 4 | 2 | 1 | 2 |
| 2021-1225 | moderate-low | Male | 70 | T4 | N0 | M0 | 2 | 3 | 6 | 1 | 3 | 3 |
| 2020-30377 | moderate-low | Male | 79 | T4 | N2 | M0 | 1 | 3 | 3 | 2 | 2 | 4 |
| 2020-30227 | moderate-low | Male | 57 | T4 | N2 | M0 | 2 | 3 | 6 | 1 | 2 | 2 |
| 2020-29939 | high | Male | 63 | T1 | N0 | M0 | 2 | 2 | 4 | 1 | 2 | 2 |
| 2020-33845 | moderate-low | Male | 67 | T4 | N2 | M0 | 2 | 2 | 4 | 2 | 1 | 2 |
| 2020-27321 | low | Male | 64 | T4 | N0 | M0 | 2 | 2 | 4 | 1 | 2 | 2 |
| 2020-20125 | low | Male | 61 | T1 | N1 | M0 | 3 | 2 | 6 | 2 | 2 | 4 |
| 2020-13882 | Ulcerated tubular adenoma | Male | 56 | T4 | N1 | M0 | 3 | 3 | 9 | 1 | 3 | 3 |
| 2020-25947 | low | Male | 68 | T4 | N2 | M0 | 2 | 2 | 4 | 1 | 2 | 2 |
| 2020-18836 | low | Male | 68 | T4 | N3 | M0 | 3 | 3 | 9 | 3 | 2 | 6 |
| 2020-25779 | low | Male | 76 | T4 | N2 | M0 | 3 | 2 | 6 | 1 | 1 | 1 |
| 2020-25382 | low | Male | 64 | T4 | N2 | M0 | 3 | 2 | 6 | 1 | 3 | 3 |
| 2020-21169 | low | Male | 66 | T4 | N3 | M0 | 3 | 2 | 6 | 2 | 2 | 4 |
| 2020-13282 | low | Male | 67 | T4 | N3 | M0 | 2 | 3 | 6 | 3 | 2 | 6 |
| 2020-6807 | low | Male | 63 | T2 | N1 | M0 | 3 | 2 | 6 | 2 | 3 | 6 |
| 2020-10339 | moderate-low | Female | 70 | T4 | N2 | M0 | 3 | 2 | 6 | 2 | 2 | 4 |
| 2020-5702 | moderate | Male | 69 | T3 | N2 | M0 | 3 | 3 | 9 | 1 | 3 | 3 |
| 2020-21122 | high | Male | 75 | T4 | N1 | M0 | 2 | 3 | 6 | 3 | 2 | 6 |
| 2020-25369 | moderate | Male | 66 | T4 | N1 | M0 | 2 | 2 | 4 | 2 | 1 | 2 |
|  |  |  |  |  |  |  | TUMOR | | | Normal tissue | | |
